# Advanced management of intermediate-high risk pulmonary embolism

**DOI:** 10.1186/s13054-021-03679-2

**Published:** 2021-08-31

**Authors:** Tatiana Weinstein, Himanshu Deshwal, Shari B. Brosnahan

**Affiliations:** Department of Pulmonary and Critical Care, New York University School of Medicine, New York, NY USA

## Abstract

This article is one of ten reviews selected from the Annual Update in Intensive Care and Emergency Medicine 2021. Other selected articles can be found online at https://www.biomedcentral.com/collections/annualupdate2021. Further information about the Annual Update in Intensive Care and Emergency Medicine is available from https://link.springer.com/bookseries/8901.

## Introduction

Pulmonary embolism is extremely common both in the general public and in hospitalized patients, but patients who have intermediate-high risk pulmonary embolism continue to pose significant treatment dilemmas. This is because the short-term mortality of a pulmonary embolus ranges from 2% in normotensive patients, 30% in patients with right ventricular (RV) dysfunction, and up to 65% in patients with cardiac arrest on presentation [1].

Understanding why a pulmonary embolism can pose such danger is anchored in the delicate balance that exists between the thrombus and obstructive shock. Once thrombus has formed in or embolized to the pulmonary artery, it acutely generates increases in pulmonary hypertension inducing right-sided heart failure. This is the primary driver of mortality in patients presenting with acute pulmonary embolism [2].

The increase in RV afterload is not simply from the physical obstruction of the pulmonary vascular bed but also the result of vasoconstrictive effects of thrombus-derived mediators such as thromboxane-A2 and serotonin [3]. Although the right ventricle dilates to overcome the rise in pulmonary vascular resistance (PVR), eventually the dilation increases to the point of myocyte dysfunction and decreased strength of RV contraction. Additionally, the pressure overload in the right ventricle results in bowing of the intraventricular septum, decreasing left ventricular (LV) preload and negatively impacting cardiac output [4].

Treatment should be modified based on disease severity, but at present no perfect predictors exist to determine which patients will decompensate [5]. Clinical decision must be made by integrating clinical evaluation, often employing a pulmonary embolism score, in conjunction with imaging and laboratory markers that note RV dysfunction and injury. This method seems to correlate best with risk of decompensation [1, 6].

**Fig. 1:**
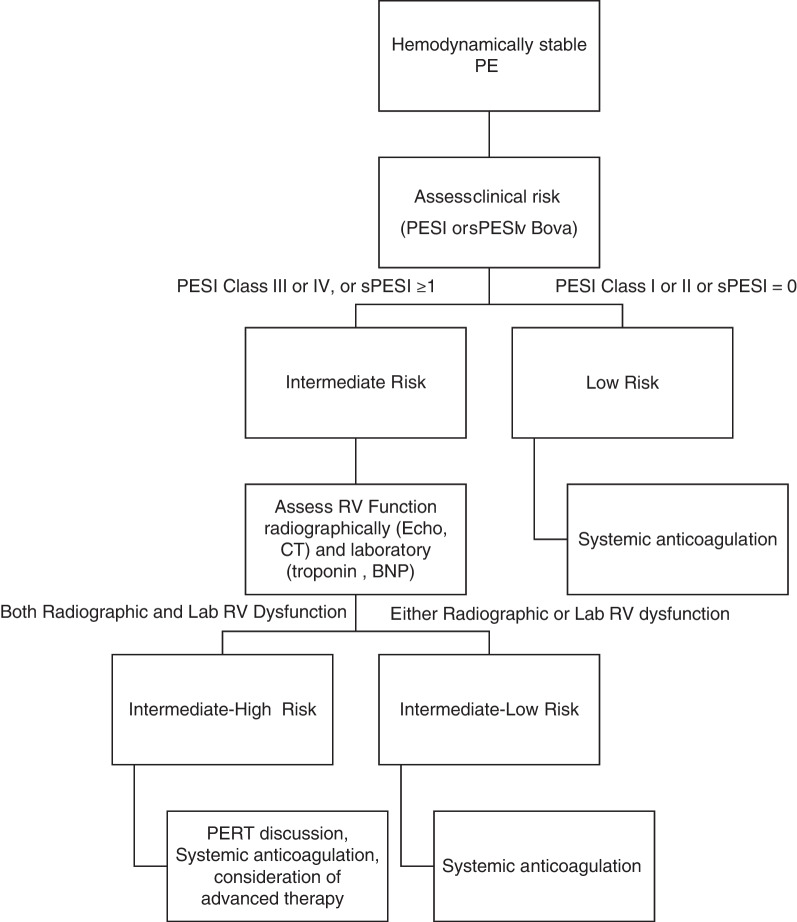
Treatment algorithm for hemodynamically stable pulmonary embolism (PE). *BNP* brain natriuretic peptide, *PESI* Pulmonary Embolism Severity Index, *sPESI* simplified PESI, *RV* right ventricular, *CT* computed tomography, *PERT* pulmonary embolism response team. ( Adapted from 2014 ESC guidelines [11])

The Bova score was developed to determine which hemodynamically stable patients with pulmonary embolism had worse outcomes. Patients with a heart rate ≥ 110 beats/min, systolic BP 90–100 mmHg for at least 15 min, RV dysfunction, and elevated cardiac troponin had an increased risk of decompensation [7, 8]. Other scores, such as the Pulmonary Embolism Severity Index (PESI) or simplified PESI (sPESI), combined with an assessment of RV function have been used to divide patients into intermediate-low risk and intermediate-high risk and help make treatment decisions [4, 9, 10]. In this chapter, we will review the evidence for various treatment modalities in patients with intermediate-high risk pulmonary embolism. Intermediate-high risk pulmonary embolism is best defined as a patient who has a pulmonary embolism, is hemodynamically stable, but has an elevated pulmonary embolism score and both radiographic and laboratory signs of right heart strain (Fig. 1) [11].

## Treatment of high-intermediate risk submassive pulmonary embolism

Once a diagnosis of pulmonary embolism is made, prompt initiation of anticoagulation is imperative as it has been shown to reduced mortality [12]. Similarly, conservative efforts to reduce the RV afterload, including oxygen supplementation and inhaled nitric oxide (iNO) to assist with pulmonary vasodilation can aid in preserving stability [13]. In cases of severe RV dysfunction, an inotropic agent such as dobutamine, should be initiated [14]. Supportive vasopressor therapy is required to keep mean arterial pressures (MAP) greater than 65 mmHg, with norepinephrine as the treatment of choice [15]. However, once dobutamine or vasopressors are used, the patient has progressed to a high-risk pulmonary embolism category and treatment algorithms can change. In significant pulmonary embolism, high afterload leads the right ventricle to dilate further. The use of extraneous intravenous fluid therapy can lead to acute RV decompensation by worsening septal shift and impacting LV preload [16]. In patients who are clinically deteriorating because of hypoxemia and respiratory distress, the decision to pursue invasive positive pressure ventilation is challenging due to concern for worsening RV afterload. The application of positive thoracic pressure can cause an acute decrease in RV preload. Therefore, an attempt to try conservative treatment, such as high flow nasal oxygen, should be considered prior to considering mechanical ventilation. If mechanical ventilation is pursued, hemodynamically neutral agents should be used for induction as the elevated PVR makes the right ventricle extremely preload sensitive. The use of propofol, a negative inotrope, has been associated with increased mortality in submassive pulmonary embolism and should be avoided [17].

### Systemic thrombolytic therapy

While the use of systemic thrombolytic therapy is recommended in high-risk pulmonary embolism, defined as hemodynamically unstable patients or in patients after the return of spontaneous circulation, application in intermediate-risk pulmonary embolism is less well defined [15]. Patients with high-risk pulmonary embolism should undergo thrombolysis, in the absence of contraindications, as thrombolysis reduces mortality by almost 50% (3.9–2.2%) compared with anticoagulation alone [18], yet this is counterbalanced by an increase in major bleeding (3.4–9.2%) and intracranial hemorrhage (0.2–1.5%). There is clear indication for use of systemic thrombolysis in the setting of rescue therapy for patients with submassive pulmonary embolism who have evidence of hemodynamic deterioration or who have not responded appropriately to anticoagulation [15]. When to use systemic thrombolysis in intermediate-risk pulmonary embolism poses a complex clinical dilemma with careful weighing of the risk–benefit ratio needed. This is because patients with intermediate-low risk pulmonary embolism will do well without escalation of therapy, and the addition of thrombolysis only adds risk without benefit. Several studies addressed below have looked at various dosages for systemic therapy with mixed results.

The European Pulmonary Embolism Thrombolysis (PEITHO) trial, the largest randomized controlled trial (RCT) to date, randomized 1004 patients with normotensive, submassive pulmonary embolism who had RV strain to weight-based tenecteplase with standard parenteral anticoagulation or parenteral anticoagulation alone [19]. Although the primary outcome was met, a decrease in the combination of death or decompensation, with decompensation making up most of the benefit, the study observed significant increases in major bleeding including intracranial hemorrhage in the tenecteplase group [19]. Nevertheless, the results solidified the role of tenecteplase in rescue therapy.

Another trial, TOPCOAT (Tenecteplase Or Placebo: Cardiopulmonary Outcomes At Three months), examined outcomes in patients with submassive pulmonary embolism randomized to low-molecular-weight heparin plus tenecteplase or placebo and demonstrated improvement in cardiopulmonary outcomes at 90 days with respect to dyspnea, quality of life scores, echocardiographic measures of RV function, and walk distance, but was not powered for mortality [20]. In meta-analysis, the stable hemodynamic subgroup has yet to show a clinically significant mortality benefit and given the increase in major bleeding, including intracranial hemorrhage, defining the exact subgroup that would benefit from more aggressive therapy remains elusive [21]. This is perhaps because intermediate-risk pulmonary embolism encompasses a large heterogeneous group of patients including those with lowintermediate risk and intermediate-high risk. The appropriate phenotyping of a patient becomes paramount when enrolling in trials to assess the true benefit of systemic thrombolysis. Because of this there remains a knowledge gap in managing patients with intermediate-high risk pulmonary embolism; currently treatment is guided mostly by a multidisciplinary and individualized approach.

Given the bleeding complications observed with conventional thrombolysis dosage, consideration was given to a ‘half-dose’ thrombolytic therapy. The rationale was that the lower dose would have the ability to maximize benefit of acutely lowering PVR while minimizing bleeding complications. The MOderate Pulmonary Embolism Treated with Thrombolysis trial, (MOPETT), was a single center study that aimed to determine whether half-dose recombinant tissue plasminogen activator (rtPA) would reduce rates of pulmonary hypertension (on echocardiogram) at 28 months. The incidence of pulmonary hypertension on echo was 57% in the anticoagulation group compared to 16% in the rtPA group and there was no increased risk of bleeding. However, the 57% incidence of pulmonary hypertension in the control arm is disparate to known historical controls [22]. Therefore, there has been little change in clinical practice based on this study. At the present time, there is no convincing evidence to support ubiquitous use of systemic thrombolysis at any dose in hemodynamically stable patients and current guidelines do not recommend its use [15, 23, 24].

While a lower dosage may be ideal for some cases of pulmonary embolism, there are questions surrounding whether this approach is equivalent to higher doses in reducing PVR. We know that systemic thrombolytic therapy in patients with high- risk pulmonary embolism can be unsuccessful, as defined by persistent clinical instability or RV dysfunction up to 36 h after therapy, and accordingly the rate of inadequate response may increase as the thrombolysis dosage is lowered [25]. Analysis of a prospective single-center registry demonstrated higher mortality and higher recurrent pulmonary embolism in patients who had repeat-dose thrombolysis compared to surgical embolectomy, with similar bleeding risk. The study also noted that bleeding events in repeat dose therapy were all fatal [25]. To add to the discussion, the timing of the thrombolytic therapy can affect efficacy; thrombolysis is known to be most effective within 48 h of thrombosis generation. Early dosing offers the greatest benefit in reducing pulmonary artery pressure and RV dilation, yet delayed use for up to 2 weeks after symptoms has also shown some benefit [26]. Therefore, all applications of systemic thrombolysis may not be equal. With the advent of catheter-directed therapies, determining when to use systemic thrombolysis in intermediate-high risk PE has become further complicated. Catheter- directed therapies use less fibrinolytics but take longer to employ than systemic fibrinolytics.

### Catheter-directed therapies

Percutaneous catheter-directed therapies offer an alternative to systemic thrombolysis, as well as a minimally invasive alternative to surgical thrombectomy for patients with high-intermediate risk pulmonary embolism at increased risk for decompensation. Several catheter-based treatment strategies have been utilized in clinical practice; however where catheter-directed therapies fit in the treatment algorithm for intermediate risk pulmonary embolism is still controversial.

Catheter-directed therapy can mean mechanical removal of clot alone or in conjunction with catheter-based thrombolysis. Mechanical therapies are good treatment options when a patient cannot tolerate fibrinolysis but has a physiologically significant thrombosis. The location of the clot dictates the utility of catheter-directed therapies as the pulmonary embolus must be proximal for the therapy to be effective. While there are larger vacuum-based therapies that require placement on extra-corporeal oxygenation prior to their use, we will not review those as they would be unlikely to be used in intermediate-risk pulmonary embolism. Rheolytic thrombectomy, with devices such as AngioJet^®^, removes the thrombus by injecting a saline jet from the distal port under a high-pressure, thus creating a negative pressure force, while a separate catheter helps evacuate the thrombus [27]. Rheolytic thrombectomy has become less popular, because as the thrombus breaks down there can sometimes be a sudden release of adenosine causing hemodynamic decompensation mostly evidenced by hypotension and bradycardia [28].

The FlowTriever^®^ device has a suction catheter alongside three nitinol mesh disks that help remove residual clot after the initial thrombus is removed using suction. The advantage of the FlowTriever^®^ device is that it offers a complete evacuation of proximal thrombi. The nitinol mesh disks are available in several sizes, allowing the proceduralist to choose an optimal size for each patient. The FLARE study demonstrated that the FlowTriever^®^ results in significant improvement in the right ventricle to left ventricle ratio at 48 h. While no major bleeding or deaths were noted, the adverse event rate was 3.8%, including one pulmonary hemorrhage and three procedure-related clinical deteriorations [29]. The clinical use of FlowTriever^®^ is often limited by the blood removed with the thrombus.

Mechanical catheter-directed therapy can be used in conjunction with catheter-directed thrombolysis or thrombolysis can occur on its own. Catheter-directed thrombolysis is when low-dose fibrinolytic agents are directly injected into the pulmonary artery at a slow infusion rate, often over the course of 12–24 h. The theoretical advantage of this technique is that the fibrinolytic infusion is at the site of thrombosis and a lower dose of fibrinolytics can be given despite longer exposure. There is conflicting evidence as to whether catheter-directed thrombolysis has less bleeding risk compared to systemic thrombolysis [30, 31].

Catheter-based therapies can help normalize the pressure in the right side of the heart more quickly than anticoagulation. The ULTIMA (ULTrasound Accelerated ThrombolysIs of PulMonAry Embolism) trial reported improvement in the right ventricle to left ventricle dimension ratio when ultrasound-assisted catheter-directed thrombolysis was used with unfractionated heparin compared to unfractionated heparin alone [32]. Although the study included only 59 patients, only three minor bleeding complications were noted in the ultrasound-assisted catheter-directed thrombolysis group compared to one in the heparin-only group. Similar results of reduced RV strain by decreasing pulmonary artery pressure, and no major bleeding complications were noted in two prospective single-armed studies: SEATTLE-II (A Prospective, Single-arm, Multi-center Trial of EkoSonic^®^ Endovascular System and Activase for Treatment of Acute Pulmonary Embolism) and PERFECT (Pulmonary Embolism Response to Fragmentation, Embolectomy, & Catheter Thrombolysis) trials [33, 34]. The OPTALYSE (Optimum Duration of Acoustic Pulse Thrombolysis Procedure in Acute Intermediate-Risk Pulmonary Embolism) trial reported that a shorter duration of 6–12 h of ultrasound-guided assisted catheter-directed thrombolysis with a lower dose of the fibrinolytic agent was also able to improve RV strain and decrease in RV afterload compared to longer durations and higher doses [35]. Given these studies, it seems like the optimal patient for catheter-directed therapies would be an intermediate risk patient with pulmonary embolism who is on the cusp of hemodynamic compromise; however, identification of this cohort remains a challenge.

The field of catheter-based treatment of intermediate-high risk pulmonary embolism continues to evolve. Its utility depends on the availability of an expert proceduralist and the institution’s access to use of an extracorporeal bypass circuit in the case of hemodynamic decompensation. The benefits, risks, and alternatives of the selected procedure must be discussed in a multidisciplinary manner and with the patient to improve outcomes and minimize complications.

## Pulmonary embolism response teams

The complexity of managing patients with intermediate-high risk pulmonary embolism calls for a multidisciplinary approach to decision-making as care may have to be individualized on a case-by-case basis. While echocardiography, biomarkers, and risk-stratification strategies help in decision making, it often becomes challenging to predict outcome in patients with intermediate-high risk pulmonary embolism. Institutions have developed pulmonary embolism response teams (PERTs) to assist in treatment strategies and the possible need for advanced therapies such as fibrinolysis versus catheter-based treatment versus surgical embolectomy on a case-by-case basis. While the composition of each team varies among institutions, they most often include some variation of pulmonologists, thoracic surgeons, cardiologists, interventional radiologists, and intensivists. It remains to be known whether PERTs improve outcomes, but they offer the best opportunity for a multidisciplinary approach to managing pulmonary embolism. The 2019 ESC guideline recommends forming an interdisciplinary team, such as a PERT, if resources are available [15]. Given the lack of hemodynamic predictors of these patients, we believe that PERTs represent the best method to weigh risk and benefit for each treatment option in patients not only with intermediate-high risk pulmonary embolism but also other pulmonary embolism conundrums.

## Submassive pulmonary embolism: rescue therapy

### Surgical embolectomy

Surgical pulmonary embolectomy has classically been reserved for patients with massive pulmonary embolism who cannot receive fibrinolysis or remain unstable after administration, or for patients with intermediate-high risk pulmonary embolism who either fail thrombolysis or have an absolute contraindication [15]. Additionally, a definitive surgical approach is recommended for patients with high-risk thrombi, such as those with appreciable clot in the right heart near or through a patent foramen ovale [36, 37]. Surgical embolectomy can rapidly restore pulmonary blood flow and relieve acute obstruction. The surgical approach is through a median sternotomy and requires the patient to be placed on cardiopulmonary bypass (CPB), typically without aortic cross-clamping or cardioplegic arrest to avoid additional ischemic injury to an already stunned right ventricle. This is followed by an incision through the pulmonary trunk and the main pulmonary arteries with subsequent extraction of the acute clot [36, 38, 39]. All patients should have an echocardiogram completed pre-operatively for an assessment of right and left sided heart function, and detection of a patent foramen ovale or an atrial septal defect, which helps to understand the risk of paradoxical embolism [39].

Systemic thrombolysis and catheter-directed therapies have emerged at the forefront of management to acutely relieve RV obstruction in intermediate-high risk pulmonary embolism as surgical pulmonary embolectomy has historically been associated with a higher mortality. However, over time, surgical technique has been revised and standardized to minimize perioperative mortality and thus sparked a renewed interest in expanding the scope of surgical interventions. Recent data from experienced surgical centers have shown in-hospital mortality as low as 11.7% in patients undergoing surgical embolectomy for acute high-risk pulmonary embolism. On deeper review of the data, it was noted that this value was largely driven by patients with massive pulmonary embolism and those with pre-operative arrest rather than patients in the intermediate-high risk pulmonary embolism category [40]. The safety of surgical embolectomy in submassive pulmonary embolism has been further underscored in other small single center studies, with one such study quoting no mortality in their cohort of patients [41]. In fact, the mortality from surgical pulmonary embolectomy in acute pulmonary embolism has been shown to be equivalent to that from thrombolysis. The New York State Registry, which included 174,322 patients hospitalized with pulmonary embolism between 1999 and 2013, revealed no difference in short-term mortality between surgical embolectomy and thrombolysis. Moreover, those who underwent surgical pulmonary embolectomy had lower rates of stroke, recurrent pulmonary embolism, and need for reintervention [42]. Improvements in operative techniques and subsequent outcomes have highlighted that surgical pulmonary embolectomy is both safe and effective. This has led to garnered interest in perhaps expanding the criteria for surgical referral, particularly in cases of intermediate-high risk pulmonary embolism [40–42]. Nevertheless, this is not yet formally part of any guideline and likely requires a multidisciplinary discussion on a case-by-case basis.

### RV assist devices

Mechanical circulatory support using RV assist devices, as a bridge to or in combination with definitive therapy has been explored in patients who face high risk of RV decompensation and circulatory collapse. The Impella device is an 11-French catheter with a 22-French pump head that is percutaneously placed under fluoroscopy through the femoral vein and advanced into the pulmonary artery. The device pulls blood from an inlet that sits in the inferior vena cava and expels it directly into the pulmonary artery. It can maintain a perfusion of 4.4 l/ min for up to 2 weeks. The device has been best studied for its use in RV failure after a LV assist device, acute myocardial infarction, heart transplant, or open-heart surgery [43]. The data on use in acute pulmonary embolism are limited. Small retrospective case series have supported its use in high-intermediate risk and massive pulmonary embolism in terms of hemodyamic benefits, survival, and recovery of RV function after device extraction [44]. Despite these appealing benefits, at present it has not been approved for use in RV failure in the setting of acute pulmonary embolism and is therefore not included in the current guidelines.

### Extracorporeal membrane oxygenation

In patients who are hemodynamically compromised from an acute pulmonary embolism, venoarterial extracorporeal membrane oxygenation (ECMO VA-ECMO) can be considered as an alternative means of circulatory support. Current guidelines recommend utilization of ECMO as a means of mechanical support for patients with acute high-risk pulmonary embolism and refractory shock with the caveat that it is used in combination with definitive therapy, such as surgical embolectomy or catheter-directed therapy [15]. Additionally, ECMO may be helpful in the setting of cardiac arrest, though again, only as a bridge to definitive therapy [15].

There is currently a paucity of data surrounding ECMO as stand-alone therapy with anticoagulation and, thus, it is not a guideline-supported therapeutic avenue [45]. The data regarding ECMO in acute pulmonary embolism are limited to case reports and series, as well as smaller observational studies that are subject to variable bias [15, 45]. There are no RCTs assessing its use and limited information regarding outcomes. One recent review of 78 patients collected from case reports and series reported a 70% survival in patients using ECMO in massive and submassive pulmonary embolism. The authors further noted that this survival benefit was not associated with any one definitive treatment modality. Poorer outcomes were noted in patients where ECMO was instituted whilst in cardiopulmonary arrest and worsened further if initiated greater than 30 min from the time of arrest [45]. This suggests that, in the right patient and if initiated early, ECMO is a potentially life-saving therapeutic option that can provide clinical stability to allow for definitive therapy. However, it is important to underscore that ECMO is associated with a high incidence of complications, such as bleeding and infection, and outcomes are largely dependent on the experience of the center and patient selection [15].

## Conclusion

While the optimal management of patients in the intermediate-high risk pulmonary embolism group remains to be defined, a combination of clinical variables, biomarkers and imaging studies may assist in identifying those patients that are most likely to benefit from a closely monitored setting [6, 18]. At present there are a large number of treatment options ranging from various thrombolysis dosages, catheter-directed therapies, surgical therapies, and peripherally inserted devices that can aid in augmenting cardiac output. Developing a superior method to determine who in the intermediate risk group would benefit remains paramount to investigating and further defining treatment for this group. While it is clear that there is benefit in aggressive treatment in the patient who needs it, if patients are not at true risk for decompensation then aggressive treatment only comes with more risk. It is perhaps not the treatment of pulmonary embolism that needs defining but rather better individualized hemodynamic monitoring and prediction of decompensation that hold the key. Until a better characterization of this population can be made we assert that the best treatment intervention may be the implementation of PERTs so that an educated discourse can be made on a case by case basis.

## Data Availability

Not applicable.

## References

[1] Jimenez D, Lobo JL, Barrios D, Prandoni P, Yusen RD (2016). Risk stratification of patients with acute symptomatic pulmonary embolism. Intern Emerg Med.

[2] McIntyre KM, Sasahara AA (1971). The hemodynamic response to pulmonary embolism in patients without prior cardiopulmonary disease. Am J Cardiol.

[3] Smulders YM (2000). Pathophysiology and treatment of haemodynamic instability in acute pulmonary embolism: the pivotal role of pulmonary vasoconstriction. Cardiovasc Res.

[4] Marcus JT, Gan CT, Zwanenburg JJ, Boonstra A, Allaart CP, Götte MJ, Vonk-Noordegraaf A (2008). Interventricular mechanical asynchrony in pulmonary arterial hypertension: left-to-right delay in peak shortening is related to right ventricular overload and left ventricular underfilling. J Am Coll Cardiol.

[5] Barco S, Konstantinides SV (2017). Risk-adapted management of pulmonary embolism. Thromb Res.

[6] Barrios D, Yusen RD, Jimenez D (2017). Risk stratification for proven acute pulmonary embolism: what information is needed?. Semin Respir Crit Care Med.

[7] Fernandez C, Bova C, Sanchez O, Prandoni P, Lankeit M, Konstantinides S (2015). Validation of a model for identification of patients at intermediate to high risk for complications associated with acute symptomatic pulmonary embolism. Chest.

[8] Bova C, Sanchez O, Prandoni P, Lankeit M, Konstantinides S, Vanni S (2014). Identification of intermediate-risk patients with acute symptomatic pulmonary embolism. Eur Respir J.

[9] Jiménez D, Aujesky D, Moores L, Gómez V, Lobo JL, Uresandi F (2010). Simplification of the Pulmonary Embolism Severity Index for prognostication in patients with acute symptomatic pulmonary embolism. Arch Intern Med.

[10] Jiménez D, Yusen RD, Otero R, Uresandi F, Nauffal D, Laserna E (2007). Prognostic models for selecting patients with acute pulmonary embolism for initial outpatient therapy. Chest.

[11] Konstantinides SV, Torbicki A, Agnelli G, Danchin N, Fitzmaurice D, Galiè N (2014). 2014 ESC guidelines on the diagnosis and management of acute pulmonary embolism. Eur Heart J.

[12] Smith SB, Geske JB, Maguire JM, Zane NA, Carter RE, Morgenthaler TI (2010). Early anticoagulation is associated with reduced mortality for acute pulmonary embolism. Chest.

[13] Kline JA, Puskarich MA, Jones AE, Mastouri RA, Hall CL, Perkins A (2019). Inhaled nitric oxide to treat intermediate risk pulmonary embolism: a multicenter randomized controlled trial. Nitric Oxide.

[14] Jardin F, Genevray B, Brun-Ney D, Margairaz A (1985). Dobutamine: a hemodynamic evaluation in pulmonary embolism shock. Crit Care Med.

[15] Konstantinides SV, Meyer G, Becattini C, Bueno H, Geersing GJ, Harjola VP (2020). 2019 ESC Guidelines for the diagnosis and management of acute pulmonary embolism developed in collaboration with the European Respiratory Society (ERS): the Task Force for the diagnosis and management of acute pulmonary embolism of the European Society of Cardiology (ESC). Eur Heart J.

[16] Ghignone M, Girling L, Prewitt RM (1984). Volume expansion versus norepinephrine in treatment of a low cardiac output complicating an acute increase in right ventricular afterload in dogs. Anesthesiology.

[17] Manchec B, Liu B, Tran T, Zuchowski C, Guruvadoo K, Parente R (2019). Sedation with propofol during catheter-directed thrombolysis for acute submassive pulmonary embolism is associated with increased mortality. J Vasc Interv Radiol.

[18] Becattini C, Agnelli G (2016). Risk stratification and management of acute pulmonary embolism. Hematol Am Soc Hematol Educ Program.

[19] Meyer G, Vicaut E, Danays T, Agnelli G, Becattini C, Beyer-Westendorf J (2014). Fibrinolysis for patients with intermediate-risk pulmonary embolism. N Engl J Med.

[20] Kline JA, Nordenholz KE, Courtney DM, Kabrhel C, Jones AE, Rondina MT (2014). Treatment of submassive pulmonary embolism with tenecteplase or placebo: cardiopulmonary out-comes at 3 months: multicenter double-blind, placebo-controlled randomized trial. J Thromb Haemost.

[21] Marti C, John G, Konstantinides S, Combescure C, Sanchez O, Lankeit M (2015). Systemic thrombolytic therapy for acute pulmonary embolism: a systematic review and meta-analysis. Eur Heart J.

[22] Sharifi M, Bay C, Skrocki L, Rahimi F, Mehdipour M, Investigators M (2013). Moderate pulmonary embolism treated with thrombolysis (from the “MOPETT” Trial). Am J Cardiol.

[23] Kearon C, Akl EA, Ornelas J, Blaivas A, Jimenez D, Bounameaux H (2016). Antithrombotic therapy for VTE disease: CHEST guideline and expert panel report. Chest.

[24] Kiser TH, Burnham EL, Clark B, Ho PM, Allen RR, Moss M (2018). Half-dose versus full-dose alteplase for treatment of pulmonary embolism. Crit Care Med.

[25] Meneveau N, Séronde MF, Blonde MC, Legalery P, Didier-Petit K, Briand F (2006). Management of unsuccessful thrombolysis in acute massive pulmonary embolism. Chest.

[26] Daniels LB, Parker JA, Patel SR, Grodstein F, Goldhaber SZ (1997). Relation of duration of symptoms with response to thrombolytic therapy in pulmonary embolism. Am J Cardiol.

[27] Song XJ, Liu ZL, Zeng R, Liu CW, Ye W (2019). The efficacy and safety of angiojet rheolytic thrombectomy in the treatment of subacute deep venous thrombosis in lower extremity. Ann Vasc Surg.

[28] Kuo WT, Gould MK, Louie JD, Rosenberg JK, Sze DY, Hofmann LV (2009). Catheter-directed therapy for the treatment of massive pulmonary embolism: systematic review and meta-analysis of modern techniques. J Vasc Interv Radiol.

[29] Tu T, Toma C, Tapson VF, Adams C, Jaber WA, Silver M (2019). A prospective, single-arm, multicenter trial of catheter-directed mechanical thrombectomy for intermediate-risk acute pulmonary embolism: the FLARE Study. JACC Cardiovasc Interv.

[30] Geller BJ, Adusumalli S, Pugliese SC, Khatana SAM, Nathan A, Weinberg I (2020). Outcomes of catheter-directed versus systemic thrombolysis for the treatment of pulmonary embolism: a real-world analysis of national administrative claims. Vasc Med.

[31] Mullan CW, Newman J, Geib M, Pichert MD, Saffarzadeh A, Hartman A (2020). Modern treatment trends and outcomes of pulmonary embolism with and without hemodynamic significance. Ann Thorac Surg.

[32] Kucher N, Boekstegers P, Müller Oliver J, Kupatt C, Beyer-Westendorf J, Heitzer T (2014). Randomized, controlled trial of ultrasound-assisted catheter-directed thrombolysis for acute intermediate-risk pulmonary embolism. Circulation.

[33] Piazza G, Hohlfelder B, Jaff MR, Ouriel K, Engelhardt TC, Sterling KM (2015). A Prospective, single-arm, multicenter trial of ultrasound-facilitated, catheter-directed, low-dose fibrinolysis for acute massive and submassive pulmonary embolism: the SEATTLE II Study. JACC Cardiovasc Interv.

[34] Kuo WT, Banerjee A, Kim PS, DeMarco FJ, Levy JR, Facchini FR (2015). Pulmonary embolism response to fragmentation, embolectomy, and catheter thrombolysis (PERFECT): initial results from a prospective multicenter registry. Chest.

[35] Tapson VF, Sterling K, Jones N, Elder M, Tripathy U, Brower J (2018). A Randomized trial of the optimum duration of acoustic pulse thrombolysis procedure in acute intermediate-risk pulmonary embolism: the OPTALYSE PE Trial. JACC Cardiovasc Interv.

[36] Iaccarino A, Frati G, Schirone L, Saade W, Iovine E, D’Abramo M (2018). Surgical embolectomy for acute massive pulmonary embolism: state of the art. J Thorac Dis.

[37] Reardon PM, Yadav K, Hendin A, Karovitch A, Hickey M (2019). Contemporary management of the high-risk pulmonary embolism: the clot thickens. J Intensive Care Med.

[38] Neely RC, Byrne JG, Gosev I, Cohn LH, Javed Q, Rawn JD (2015). Surgical embolectomy for acute massive and submassive pulmonary embolism in a series of 115 patients. Ann Thorac Surg.

[39] Greelish JP, Leacche M, Solenkova NS, Ahmad RM, Byrne JG (2011). Improved midterm outcomes for type A (central) pulmonary emboli treated surgically. J Thorac Cardiovasc Surg.

[40] Keeling WB, Sundt T, Leacche M, Okita Y, Binongo J, Lasajanak Y (2016). Outcomes after surgical pulmonary embolectomy for acute pulmonary embolus: a multi-institutional study. Ann Thorac Surg.

[41] Pasrija C, Kronfli A, Rouse M, Raithel M, Bittle GJ, Pousatis S (2018). Outcomes after surgical pulmonary embolectomy for acute submassive and massive pulmonary embolism: a single-center experience. J Thorac Cardiovasc Surg.

[42] Lee T, Itagaki S, Chiang YP, Egorova NN, Adams DH, Chikwe J (2018). Survival and recurrence after acute pulmonary embolism treated with pulmonary embolectomy or thrombolysis in New York State, 1999 to 2013. J Thorac Cardiovasc Surg.

[43] Anderson M, Morris DL, Tang D, Batsides G, Kirtane A, Hanson I (2018). Outcomes of patients with right ventricular failure requiring short-term hemodynamic support with the Impella RP device. J Heart Lung Transplant.

[44] Elder M, Blank N, Kaki A, Alraies MC, Grines CL, Kajy M (2018). Mechanical circulatory support for acute right ventricular failure in the setting of pulmonary embolism. J Interv Cardiol.

[45] Yusuff HO, Zochios V, Vuylsteke A (2015). Extracorporeal membrane oxygenation in acute massive pulmonary embolism: a systematic review. Perfusion.

